# Clinicopathological profile and management of thyroid carcinoma: a Sub-Saharan country experience

**DOI:** 10.1186/s13044-023-00173-5

**Published:** 2023-08-25

**Authors:** Moawia Mohammed Ali Elhassan, Mohamed Dafalla Awadalla Gismalla, Sahar Abdelrahman Hamid Mohamed, Areeg Faggad

**Affiliations:** 1 Department of Oncology, National Cancer Institute – University of Gezira, Wad Medani, Sudan; 2 Department of Surgery, Faculty of Medicine, University of Gezira, Wad Medani, Sudan; 3 Ministry of Health, Gezira State, Wad Medani, Sudan; 4 Department of Molecular Biology, National Cancer Institute – University of Gezira, Wad Medani, Sudan

**Keywords:** Thyroid cancer, Follicular carcinoma, Anaplastic carcinoma, Goiter, Radioactive iodine, Sudan

## Abstract

**Background:**

In Sudan, there is limited knowledge on the epidemiology, clinical characteristics and pathological patterns of thyroid cancer. To address this shortcoming, we studied the clinical, pathological and treatment patterns of thyroid cancer at the National Cancer Institute ‒ University of Gezira (NCI-UG), Sudan.

**Methods:**

We performed a retrospective health facility–based study of patients with thyroid cancer who were treated at NCI–UG from January 2009 to December 2017.

**Results:**

A total of 139 patients with thyroid cancer were identified during the study period. Tumors were more common among women (69%). Goiter was the main presenting symptom (85%). The most common type of thyroid cancer was follicular carcinoma (41%), followed by papillary carcinoma (24%), then anaplastic carcinoma (20%). The mean age of the women was 56.3 years (SD ± 14.7), compared to 52.5 years (SD ± 16.6) for the men. The frequencies of stage I, II, III, and IV were 17%, 22%, 16%, and 45%, respectively. Different types of thyroidectomies were performed in 79% of the cases, lobectomy in 4%, and no surgery in 17%. Only 28% of the cases received radioactive iodine. Palliative chemotherapy and radiotherapy were prescribed to 17% and 37% of the cases, respectively.

**Conclusion:**

Thyroid cancer is more prevalent among women and most patients present at later stages. The dominance of follicular type suggests that the majority of this population is iodine-deficient.

**Supplementary Information:**

The online version contains supplementary material available at 10.1186/s13044-023-00173-5.

## Introduction

Thyroid cancer (TC) is the most common endocrine cancer globally, it is responsible for 586,000 cases worldwide, ranking in the ninth place for incidence in 2020 [1, 2]. In the last decades, TC incidence has increased steadily and sharply risen globally [3]. This increased incidence is most likely due to a combination of an apparent increase due to frequent use of sensitive diagnostic procedures and of a true increase, a possible consequence of increased population exposure to radiation, environmental risk factors and lifestyle changes [3–7].

The incidence of TC varies in different parts of the world depending on various factors [1, 2]. Incidence rates are higher in high-income countries (HICs) than in low‒ and middle‒income countries (LMICs) [1, 2]. The rise in incidence in HICs compared to LMIC might be a result of the improved utilization of advanced radiologic and imaging techniques leading to diagnosis of occults lesions that might otherwise have gone unnoticed [3]. Moreover, increased surveillance of the thyroid gland among the population of HICs has increased the early detection rate of TC [8].

Thyroid cancer tends to affect women more often than men and the global incidence rate in women is 3-fold higher than that in men [1]. This higher incidence of TC in women compared to men could be partly explained by the following reasons. First, female oestrogen has a possible role in the development of differentiated TC [9]. Second, women tend to undergo more thyroid examinations than men because of a higher incidence of benign thyroid disease [10–12]. Third, women during the reproductive age are generally more susceptible to healthcare than men, which could lead to additional opportunities for thyroid examination and increased the early detection rate of TC [9, 12, 13].

Cancer of thyroid gland originates from thyroid follicular (acinar) cells or para-follicular (C) cells. The majority are epithelial tumors that originate from thyroid follicular cells including: papillary carcinoma, follicular carcinoma, Hurthle cell (oncocytic) carcinoma, poorly differentiated carcinoma and anaplastic (undifferentiated) carcinoma [14]. Medullary thyroid carcinoma arises from thyroid para-follicular (C) cells. The majority of TC are differentiated thyroid carcinomas, papillary or follicular carcinoma, that are highly curable and they respond well to radioactive iodine therapy (RAI) [15, 16]. In Africa, there is a wide variation in prevalence rates of TC histologic types [17].

The management of TC requires a multidisciplinary approach and guidelines to treatment. International guidelines for management of TC assume the availability of thyroid stimulating hormone (TSH) assays, sensitive diagnostic procedures, RAI, thyroid and calcium monitoring and replacement, and reliable follow-up [16, 18]. However, in low resource settings, well-trained thyroid surgeons, nuclear medicine physician, clinical oncologist, pathologist, endocrinologists and radiologist are limited in number and tend to work in isolation in areas where they exist. Furthermore, the necessary interventions recommended regularly by international guidelines are not available or are unaffordable [19]. Hence, international guidelines for the management of TC often cannot be applied to a low resource setting.

In Sudan, there is no national cancer registry. Therefore, the true incidences of cancer remain unknown. According to GLOBACAN estimates, incidence of TC for Sudanese men and women were 1.1 per 100 000 and 3.2 per 100 000, respectively [1]. There is a limited number of hospital-based studies on TC in Sudan [20–23], and most of the researches that were done in the capital city Khartoum [20, 21] or focused on differentiated TC [22, 23]. It is important to identify the TC patterns because the therapeutic approaches and the prognosis differ accordingly. Therefore, studies from various regions in our setting are needed to provide more information on the epidemiology of TC in Sudan and treatment options available in the country. In this study, we aimed to describe demographic characteristics, histopathology pattern, clinical stage and treatment of TC in Sudan.

## Methods

### Study design

We performed a retrospective descriptive study to evaluate clinical and pathological profile and treatment of patients with TC treated at the National Cancer Institute – University of Gezira (NCI-UG), Sudan between January 2009 and December 2017.

### Patients

Inclusion criteria:

- All adult patients (older than 16-year-old) with a diagnosis of TC during the study period.

Exclusion criteria:

- Patients who had incomplete records (i.e., patients without cytology or biopsy result).
- Patients who had secondary TC.

### Data collection

Patients with a confirmed diagnosis of TC were initially identified from the oncology department records database from which the record numbers were obtained. The hospital files of the patients were then retrieved from the hospital’s archive and relevant information was collected by filling out the structured data sheet (supplementary file 1). The study investigators extracted the following variables from medical records: patient sex, patient age, presenting symptoms, mode of diagnosis, fine needle aspiration findings, histopathology findings, clinical staging according to the 7th edition of the American Joint Committee on Cancer (AJCC) Cancer Staging[24], and treatment information.

### Operational definitions

- Goitre was defined as “the visible thyroid swelling or each of the lateral lobes of thyroid glands is larger than terminal phalanges of the thumb of the physician examining the patients”.
- Thyroid nodule was defined as “a discrete lesion within the thyroid gland that is radiologically distinct from the surrounding thyroid parenchyma”.
- The malignant fine needle aspiration cytology (FNAC) was defined according to the Bethesda System for Reporting Thyroid Cytopathology [25].
- Total thyroidectomy was defined as “the surgical removal of the whole thyroid gland“ [26].
- Near-total thyroidectomy was defined as “the surgical removal of both thyroid lobes except for a small amount of thyroid tissue (on one or both sides less than 1.0 mL)” [26].
- Subtotal thyroidectomy was defined as “the surgical removal of both thyroid lobes except 3 g to 5 g on the less affected side of the thyroid gland” [26].
- Thyroid lobectomy was defined as “the surgical removal of one thyroid lobe” [27, 28].

### Statistical analysis

The data was coded, entered and analyzed by Statistical Package for Social Science (SPSS Corporation, Chicago, IL, USA); version 24. We present statistical data as frequencies and percentages for categorical variables, or as mean and SD for numerical variables. The statistical analysis of the differences between the groups was performed by X^2^ test. A P-value less than 0·05 was considered significant.

### Ethical considerations

Ethical approval for this study was obtained from the ethics committee at the National Cancer Institute ‒ University of Gezira (NCI-UG/REC: 2021-017).

## Results

**Fig. 1:**
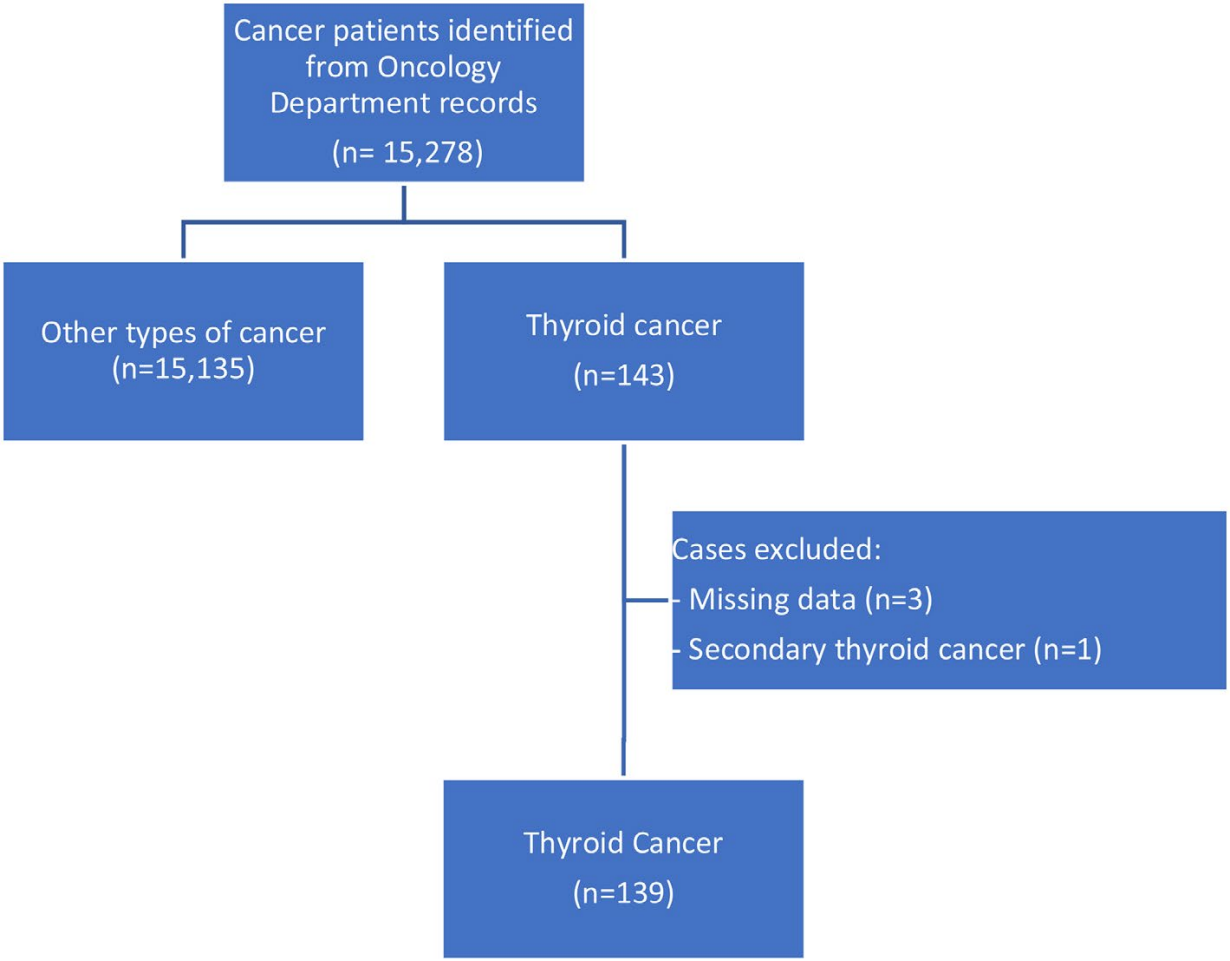
A flow chart describing the process applied to identify the eligible patients to be enrolled in the study

Out of 15 278 adults’ cases of cancer recorded at the NCI-UG during the period of the study, a total of 143 (0.9%) patients were diagnosed with TC. Out of them, 139 patients met the criteria for inclusion in this study (Fig. 1). The median number of TC cases diagnosed per year was 13 (range 7–23 cases).

### Patients’ characteristics

**Fig. 2:**
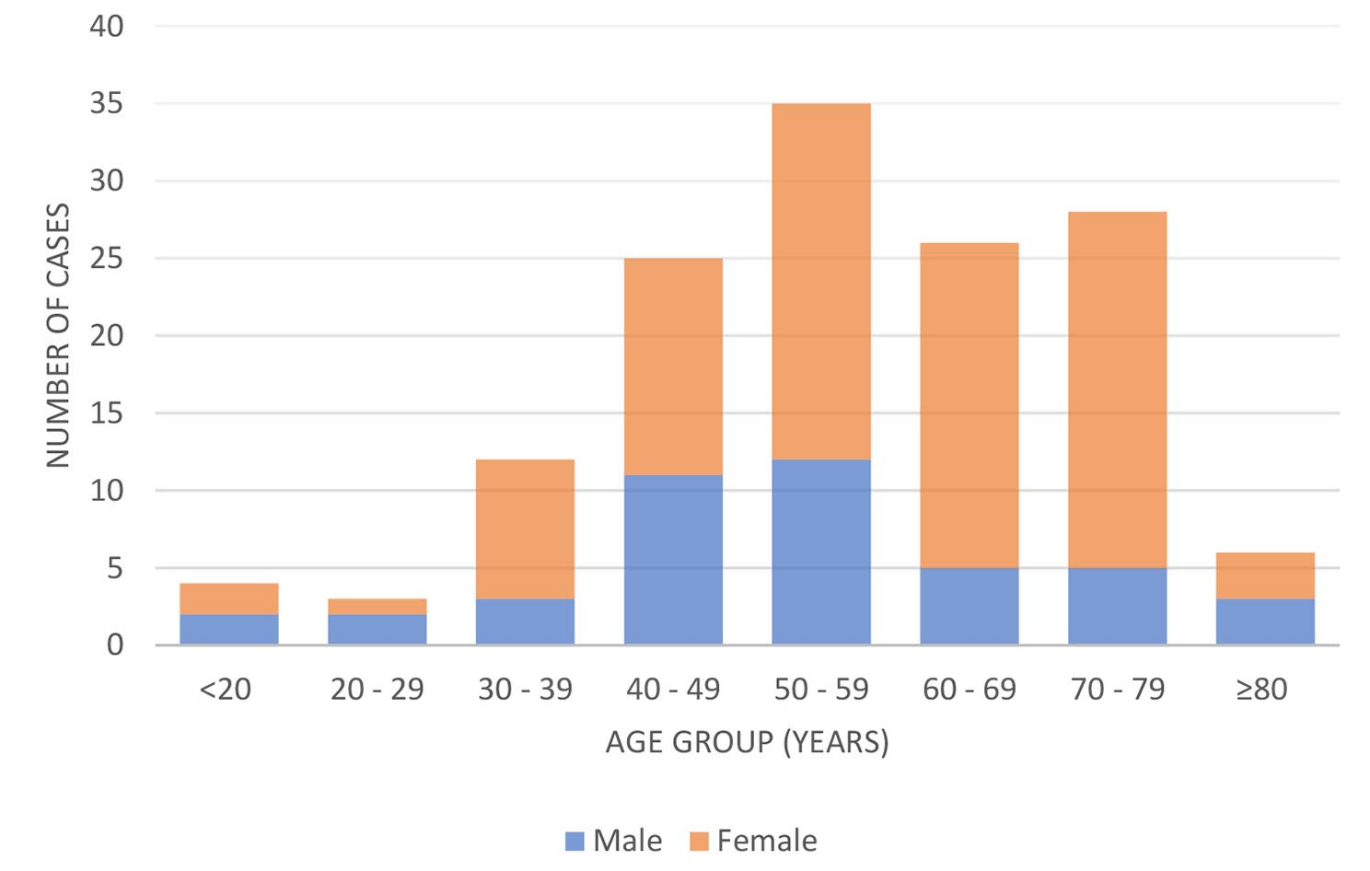
Age characteristics of patients diagnosed with thyroid cancers (n = 139)

Of the 139 patients with TC included in this study, 43 (31%) were males and 96 (69%) were females, with a female to male ratio of about 2:1. The mean age at diagnosis was 55.2 years (SD, 15.2 years). The peak age at diagnosis was earlier for men (52.2 years) than for women (56.3 years). The distribution of TC cases by age group, and sex is shown in Fig. 2.

### Presenting symptoms

Anterior neck swelling was the most frequently reported clinical presentation (n = 111, 85%), followed by bone pain (n = 13, 10%), difficulty in swallowing (n = 7, 5%) and forehead swelling (n = 4, 3%). Approximately 2% of the cases reported more than one of the stated presenting symptoms.

### Tumor characteristics

**Fig. 3:**
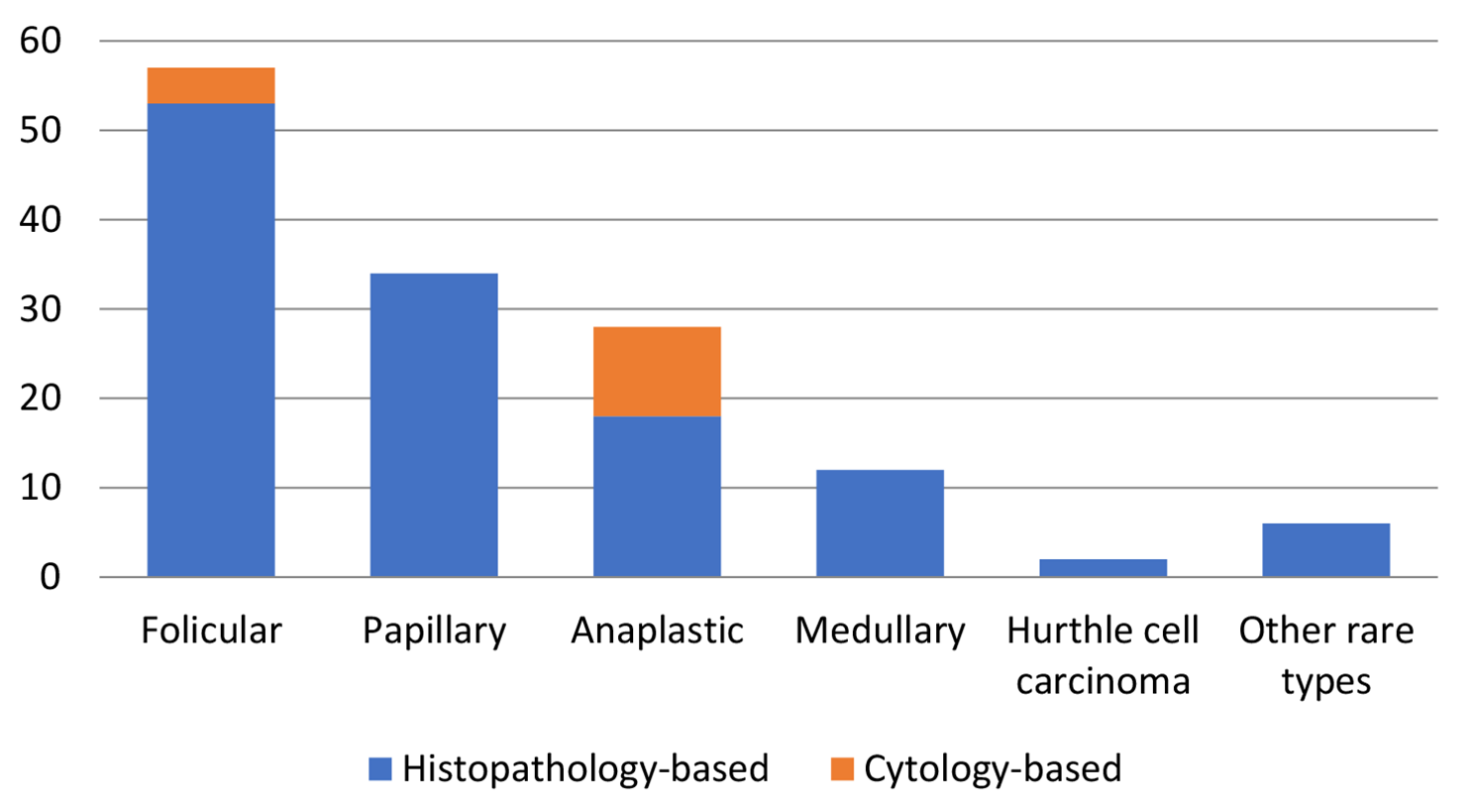
Distribution of thyroid cancers cases according to mode of diagnosis (n = 139)

**Fig. 4:**
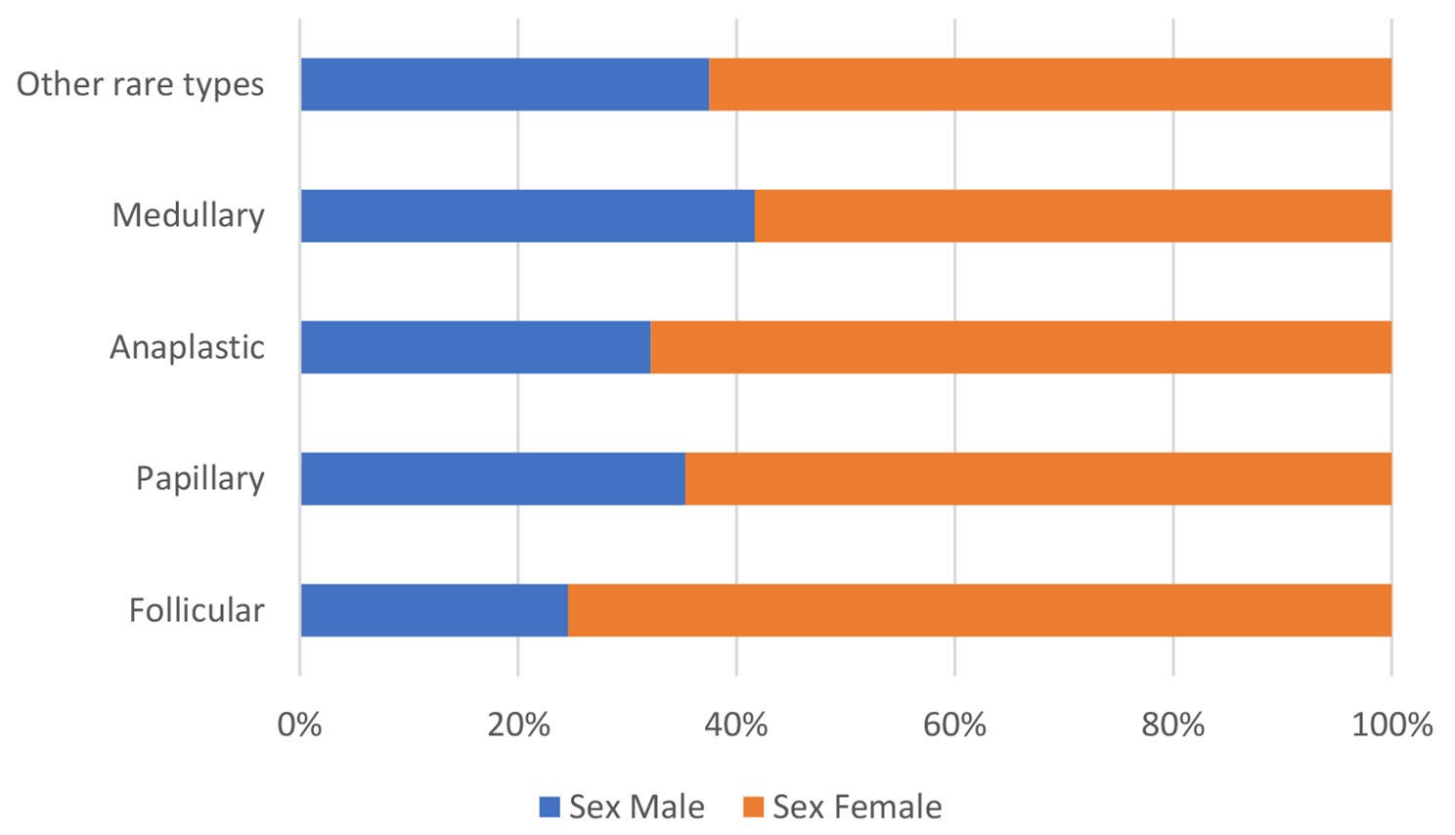
Histopathological types of thyroid cancer cases by sex (n = 139)

The majority (n = 125, 90%) of TC cases was histo-pathologically-confirmed; a small number (n = 14, 10%) of all diagnoses was based on clinical and cytology findings only (Fig. 3). Notably, most of the cytology-based TC diagnoses were anaplastic histology (n = 10). Differentiated TC accounted for 66% of all cases, followed by anaplastic (20%), medullary (9%) and other rare types (5%). The peak age at diagnosis was later for anaplastic (mean age was 64.1 years) than for other histopathology types (48.3–55.1 years) as shown in Table 1. Among all histopathologic types of TC, follicular carcinoma was the most common type (41%, n = 57 patients) as shown in Table 1, with incidence rate of 54% (n = 43) and 32% (n = 14) for the subgroups of female and male patients, respectively. Females were also predominant in all histological types and clinical stages as shown in Table 2; Fig. 4. Association of sex with histopathology types, clinical stage and lymph node metastasis are shown in Table 3.

**Table 1:** Mean age of thyroid cancer cases according to histopathology types (n = 139)

Histopathology	Frequency		Age (Years)	
	n	(%)	Mean	(SD)
Follicular	57	(41%)	53.5	(15.6)
Papillary	34	(25%)	53.4	(13.7)
Anaplastic	28	(20%)	64.1	(11.9)
Medullary	12	(9%)	48.3	(13.7)
Other rare types	8	(5%)	55.1	(14.2)
Total	139	(100%)	55.2	(15.2)

**Table 2:** Distribution of thyroid cancer cases by clinical stage, age, and sex (n = 139)

Stage	Frequency		Age (Years)		Sex	
	n	(%)	Mean	(SD)	M	F
I	23	(17%)	37.52	(11.82)	8 (19%)	15 (16%)
II	30	(22%)	59.63	(9.95)	7(16%)	23 (24%)
III	23	(16%)	51.87	(12.77)	10(23%)	13 (13%)
IV	63	(45%)	60.65	(14.21)	18(42%)	45 (47%)
Total	139	(100%)	55.2	(15.2) *	43 (100%)	96 (100%)

SD, standard deviation; M, Male; F, Female; *Mean age (SD) of the study population

**Table 3:** Association of sex with histopathology types, clinical stage and lymph node metastasis (n = 139)

	Sex		P value
	Female	Male	
Histopathology			
Follicular	43 (45%)	14 (32%)	
Papillary	22 (23%)	12 (28%)	
Anaplastic	19 (20%)	9 (21%)	0.6
Medullary	7 (7%)	5 (12%)	
Other rare types	5 (5%)	3 (7%)	
Total (%)	96 (100%)	43 (100%)	
Stage			
I	15 (16%)	8 (19%)	
II	23 (24%)	7(16%)	
III	13 (13%)	10(23%)	0.4
IV	45 (47%)	18(42%)	
Total (%)	96 (100%)	43 (100%)	
L.N metastasis			
No	80(83%)	29 (67%)	
Yes	16 (17%)	14 (33%)	0.6
Total	96 (100%)	43 (100%)	

### Clinical stage

At presentation, the majority (n = 63, 45%) of the patients had stage IV disease. Stage I, II and III were reported in 17% (n = 23), 22% (n = 30) and 16% (n = 23), respectively (Table 2). According to AJCC, all cases of anaplastic TC (n = 28, 20%) were considered as stage IV, while patient with differentiated TC younger than 45 years with distant metastasis (n = 5, 4%) were considered as stage II. The average age of patients with stage IV (60.65 years) was older than stage I (37.52 years), stage II (59.63) and stage III (51.97 years) as shown in Table 2. Out of 54 distant metastatic sites, bone was the most common site (n = 21, 39%), followed closely by lung (n = 19, 35%), then liver (n = 9, 17%), and brain (n = 5, 9%). Approximately two-thirds of patients with follicular carcinoma had distant metastases. While less than half (43%) of anaplastic carcinoma and only 18% of papillary carcinoma had distant metastases (Table 4). Multiple metastatic sites were reported in approximately 10% (5/54) of patients with distant metastasis. Less than quarter (n = 30, 22%) of patients had lymph node involvement.

**Table 4:** Distribution of Patients with Metastatic Thyroid Cancers According to Sex and Histopathology

	Site(s) of metastasis								Total
	Bone	Lung	liver	brain	bone + lung	bone + brain	liver + lung	lung + brain	
Gender									
Female	16	13	5	3	1	1	1	0	40
Male	2	4	3	0	1	0	0	1	11
Histopathology									
Follicular	9	14	2	1	2	1	0	0	29
Anaplastic	5	2	3	1	0	0	0	1	12
Papillary	2	1	2	1	0	0	0	0	6
Medullary	1	0	1	0	0	0	0	0	2
Other rare types	2	0	0	0	0	0	0	0	2

### Treatment

The treatment modalities used for the management of the 139 patients diagnosed with TC are summarized in Table 5. Total or near total thyroidectomy was the most common type of surgery, performed in 79% of the patients, while lobectomy was performed in 4%, whereas in the total cohort, tumors were found not resectable in 24 (17%) patients. All patients underwent surgical resection without being discussed in a multidisciplinary team (MDT) meeting. Systemic chemotherapy was prescribed to 24 (17%) patients. Overall, 51 (37%) patients received external beam radiotherapy (EBRT). Among these patients, 25 patients received EBRT to the neck, with ten having anaplastic TC, six follicular TC, five papillary TC and four were medullary TC. Meanwhile, the remaining 26 patients received EBRT to sites of distant metastasis, with 20 having follicular TC, four anaplastic TC and two papillary TC. Out of all cases received palliative EBRT to metastatic site, 21 patients received palliative EBRT to bone metastasis while only five patients received whole brain EBRT to brain metastasis. Only 39 (28%) patients received RAI.

**Table 5:** Types of treatment (n = 139 patients)

Type of treatment	n	%
**Type of Surgery**		
	78	57%
Total Thyroidectomy	26	19%
Thyroidectomy with LND	4	3%
lobectomy + isthmectomy	6	4%
No surgery	24	17%
**External Beam RT**		
No RT	88	63%
Neck RT	25	18%
Bone RT	21	15%
Whole Brain RT	5	4%
**Chemotherapy**		
No chemotherapy	115	83%
Palliative chemotherapy	24	17%
**Radioactive Iodine (RAI)**		
No	100	72%
Yes	39	28%

LND, Lymph node dissection; RT, Radiotherapy

## Discussion

During the period under review, approximately 0.9% of cancer cases treated at NCI-UG were TC. The findings of the present study show that follicular carcinoma was the most common type of TC, followed in descending order by papillary carcinoma, anaplastic carcinoma and medullary carcinoma. The female to male ratio was 2: 1. Most of the patients had extraglandular spread with nodal (21.5%) or distant metastases (36.7%) at the time of diagnosis. Patients with follicular carcinoma had a higher incidence of distant metastases than other pathological types. Approximately 80% of the cases had total thyroidectomy and only 28% received adjuvant RAI.

The incidence rates of TC are more than 2-fold higher in HICs compared to LMICs, and differences in TC diagnostic practices are likely the major factor for these differences [19]. Other factors include variations in exposure to unknown and known risk factors and the quality of cancer registration [28]. Yet, there is limited information on the situation of TC in Sudan. Understanding the pattern and burden of TC in the country is crucial for policy maker to develop effective diagnostic and therapeutic approaches that are cost-effective and appropriate.

We found that TC represents less than 1% of new cancer cases during the study period, consistent with the prevalence reported in the medical literature [29]. The preponderance of TC in women over men observed in this study is similar to previous studies from Sudan and the global pattern [10, 21, 30, 31]. The mean age of TC patients among our study population was 55.2 years, which is similar to a previous study from Sudan [21] and slightly older than that reported in studies from Nigeria (49 years) [32] and Cameroon (47 years) [33]. In this study, the most common age of TC affliction is reported in the fifth and sixth decades of life. Worldwide, the most common age at onset of TC for female and male patients were 15 to 49 years and 50 to 69 years, respectively [34]. Interestingly, our data showed that the peak age at diagnosis was earlier for men (52.2 years) than for women (56.3 years). The older age at diagnosis for female patients found in this study suggests that either TC was more likely to affect older women or indicate underdiagnosis, especially in women, due to lack of awareness of TC and its symptoms.

Worldwide, papillary carcinoma is reported to be the most common type of TC accounting for 85–90% of all TC cases, followed by follicular carcinoma [35]. However, in the current study, follicular carcinoma was the most frequent type (41%), followed by papillary carcinoma (24%), anaplastic carcinoma (20%) and medullary carcinoma (9%). This predominance of follicular carcinoma reported in this study is similar to previous studies from Sudan[20, 21] and prior studies on TC conducted in Southern Africa [36–38], Western Africa [39, 40], and Eastern Africa [38, 41]. However, there are studies conducted in Africa which showed papillary carcinoma as the most frequent type of TC [33, 42–45]. In the African continent, there is a wide variation in the prevalence rates of histological types of TC as follows: papillary (6.7–72.1%), follicular (4.9–68%), anaplastic (5–21.4%) and medullary (2.6–13.8%) [17]. Notably, there is a changing trend in Africa toward the more frequent occurrence of papillary carcinoma compared to follicular carcinoma; the most likely explanation for this changing trend may be attributable to the changing iodine status as a result of widespread iodization program [17, 46].

The predominance of follicular carcinoma in this study is possibly due to the higher prevalence of iodine deficiency and goiter in Sudan. In this regards, previous reports support the hypothesis of distinct causation from iodine deficiency to follicular TC [47, 48], and that residence in an endemic goiter region is associated with increased risk of follicular carcinoma [48, 49]. In Sudan, the overall prevalence of goitre is 38.8% (ranged, 12.2–77.7%), mainly due to iodine deficiency [50]. Although the iodine deficiency disorders control program in Sudan was initiated in 1989 [51], a survey in 2011 revealed insufficient iodination in schoolchildren from the capital cities of nine states. In this survey the median urinary iodine concentration (MUIC) was 65.5 µg/l [50]. A recent survey revealed that iodine status is suboptimal among Sudanese women of reproductive age [52]. According to the iodine global network, the MUIC in 2018 for women of reproductive age in Sudan (15–49 years) was 108 µg/l [53]. School-age children and pregnant women are considered having insufficient iodine intake with the MUIC of < 100 µg/L and 150 µg/L, respectively [54].

Only a small proportion, approximately 3–10%, of all TC are Medullary TC [55]. According to this study, Medullary TC accounted for 9% of all TC cases. Hurthle cell carcinoma is a rare subtype of TC make up only 1% of our study population. Consistent with this finding, a study done in Ethiopia also reported that Hurthle cell carcinoma accounted for 1.6% of the total type of TC [56].

Anaplastic TC prevalence varies globally, with a range of 1.3 to 9.8% of all TC [57–59]. The reasons of for this variation are unknown, although studies indicate that areas with iodine deficiency have a higher prevalence of anaplastic TC and suggest that coexisting differentiated TC and multinodular goiter are common in patients with anaplastic TC [60–62]. As the current study is retrospective in nature, information regarding the presence of other thyroid diseases is lacking. Therefore, it is not possible to assess the effect of these factors on the prevalence of anaplastic TC. Almost one fifth of the cases in this study had anaplastic carcinoma, which is similar to a previous study from Sudan [20]. Immunohistochemical techniques were not implemented in the present study and most anaplastic TC diagnosis was based on cytology and clinical findings. Therefore, patients with medullary TC, thyroid non-Hodgkin lymphoma, and the insular variant of follicular TC may be diagnosed incorrectly with anaplastic carcinoma. It is interesting to note that the average age at diagnosis was later for anaplastic, which is aggressive subtypes of TC, than other types of TC, suggesting that advanced age is associated with a worse prognosis. This finding is consistent with a previous report from Romania which revealed that the average age at diagnosis of TC tend to increase with decreasing degree of differentiation of carcinoma [63].

In Sudan, diagnostic techniques including radiology, cytology, and histopathology for the evaluation of thyroid nodules are costy. Additionally, the number of laboratories and hospitals providing these services is not compatible with the size of the population and are unevenly distributed across the country. Moreover, facilities that are essential for cancer staging such as computed tomographic scans, magnetic resonance imaging scans and nuclear scans are also not widely accessible. When available are often inaccessible to most uninsured patients due to health care system’s reliance on “out of pocket” payment for those without health insurance.

In the current study, approximately 41% of TC are localized (confined to the primary site), 22% have spread to regional lymph nodes, and 37% had metastasized to distant site. The patients with follicular TC frequently present with a higher occurrence of distant metastases because of the propensity of vascular invasion. Notably, most of our patients presented late with nodal or distant metastases. This in contrast to patients from HICs where the majority (65%) of cases were localized, 29% have spread to regional lymph nodes, 3% had metastasized to distant sites, and 2% with unknown stage [64]. The early diagnosis of TC in Sudan faces many obstacles, such as lack of awareness about the early symptoms of the disease and the shortage of accessible laboratory and imaging services.

Surgery is the mainstay treatment for TC. Thyroidectomy was the commonest procedures performed for thyroid lesions at our setting during the study period. Less than 5% of our study population had lobectomy with isthmectomy, this is likely due to the fact that most cases were presented with advanced tumors at presentation which make lobectomy a difficult procedure. Omeran and colleagues recommended that a high index of clinical suspicion in areas of endemic goiters such as Sudan in order that selected cases can be subjected to proper surgical intervention at an earlier stage of the disease [20]. In Sudan, it is a common practice that patients with thyroid tumors undergo surgical resection by general surgeons without being discussed in a multidisciplinary meeting. Patients are then referred to the oncology centers only after histopathology confirmation for clinical staging and further oncological treatments.

In this study, only 28% of patients received RAI treatment. This finding is similar to a previous study from Nigeria that showed only 28.6% of patient with TC had RAI treatment [65]. A systematic review of literature reported that the RAI usage in diagnosis and treatment of TC is underutilized in sub-Saharan Africa [17]. Limited availability of RAI is a particularly pressing issue given the primacy of RAI as a treatment modality in adjuvant and palliative settings for well-differentiated subtypes (papillary, follicular and Hurthle cell) of TC. In Sudan, the health care facilities that provide RAI are limited to the centers in the capital Khartoum and Shendi city. This may result in reduced access due to the high travel burden on patients and, consequently, a poor outcome.

Systemic chemotherapy has limited efficacy in patients with advanced or metastatic TC. Recently, several targeted therapies such as multikinase inhibitors, RET inhibitors and RAF kinase inhibitors were approved as first-line systemic therapy for the treatment of advanced/metastatic differentiated TC and medullary TC [66]. However, these novel therapies are not available in our limited resource setting due to the high cost. Therefore, palliative chemotherapy was used for 17% of our patients.

There is limited information on the outcome and survival of TC in Sudan. Survival outcome of TC is beyond the scope of this study. Notably, high mortality from TC is a remarkable feature of TC in most parts of Africa. The reported 5-year survival of Ugandan patients with TC is 12.5% [67]. This is in contradistinction from what obtained in developed countries where the 5-year survival for TC is very high (98.5%) [64].The very poor prognosis of African patients with TC is most likely explained by the advanced stage at presentation and lack of appropriate treatment options. In this regard, future studies are needed to assess survival outcome of TC in Sudan.

Certain limitations should be considered in the interpretation of our findings, being cross-sectional by design and that data were collected in a retrospective manner with the inherent limitations of such a design. Also, it is a single-center study, so conclusions cannot be drawn to the whole country. Nonetheless, as the NCI-UG is one of the only two referral radiotherapy and oncology center in Sudan during the study period, our cohort could be representative of Sudan. And finally, we do not have the mortality data for our study population.

Studies on the attributable risk factor for TC in Sudan is lacking. Therefore, a case control study using up-to-date epidemiological data covering more locations and data sources is required to investigate the risk factors of TC in Sudan. This would be an effective way to provide evidence to support the planning and allocation of medical care resources for TC. Furthermore, it is important to conduct prospective studies that include large number of patients representing the entire country to explain the predominance of follicular carcinoma in Sudan.

## Conclusion

Thyroid cancer is more prevalent among women and most patients present at a late stage. The majority of patients have the follicular type, which suggests that a considerable proportion of the affected population suffers from iodine deficiency. To avoid patients seeking treatment at advanced stages, it is crucial to have early detection strategy and appropriate guidelines for thyroid nodules that is tailored to available resources. Furthermore, it is essential to implement a national healthcare plan in order to improve prompt availability of necessary diagnostic tools and effective treatment methods for individuals with TC.

### Electronic supplementary material

Below is the link to the electronic supplementary material.

Supplementary Material 1: Data Collection Sheet

## Data Availability

The datasets used and/or analyzed during this study are available from corresponding author on reasonable request.

## List of abbreviations

TC: Thyroid cancer
TSH: Thyroid Stimulating Hormone
LIMCs: Low and Middle-Income Countries
HICs: High-Income Countries
NCI-UG: National Cancer Institute-University of Gezira
AJCC: American Joint Committee on Cancer
SPSS: Statistical Package for Social Science
EBRT: External beam radiotherapy
RAI: Radioactive Iodine
