# Supplementary material for: Clinicopathological profile and management of thyroid carcinoma: a Sub-Saharan country experience
Source: Thyroid Res. 2023 Aug 25;16:35. doi: 10.1186/s13044-023-00173-5 (PMC10463320; doi:10.1186/s13044-023-00173-5)
Supplement: Supplementary file 1 — Supplementary Material 1: Data Collection Sheet [file 13044_2023_173_MOESM1_ESM.docx]

| **Clinicopathological profile and management of thyroid Carcinoma**  **Data Enter Form** | | | | | | | | | | | | | | | | | | | | | | | |
| --- | --- | --- | --- | --- | --- | --- | --- | --- | --- | --- | --- | --- | --- | --- | --- | --- | --- | --- | --- | --- | --- | --- | --- |
|  |  | | | | | | | | | | | | | | | | | | | | | | |
| **Code** |  | | | | | | | | | | | | | | | | | | | | | | |
|  |  | | | | | | | | | | | | | | | | | | | | | | |
| **Age (Years)** |  | | | | | | | | | | | | | | | | | | | | | | |
|  |  | | | | | | | | | | | | | | | | | | | | | | |
| **Sex** | Male | | | | |  | | | |  | | | | | | | | Female | | | |  | |
|  |  | | | | | | | | | | | | | | | | | | | | | | |
| **Presenting symptoms** | 1. | | | | | | | | | | | | | | | | | | | | | | |
|  | 2. | | | | | | | | | | | | | | | | | | | | | | |
|  | 3. | | | | | | | | | | | | | | | | | | | | | | |
|  |  | | | | | | | | | | | | | | | | | | | | | | |
| **Mode of diagnosis** | Histopathology | | | | | | | |  | | | | | | |  | | | | | cytology | |  |
|  | Date of Diagnosis: | | | | | | | | | | | | | | | | | | | | | | |
|  |  | | | | | | | | | | | | | | | | | | | | | | |
| **Histopathology Types** |  | | | | | | | | | | | | | | | | | | | | | | |
|  |  | | | | | | | | | | | | | | | | | | | | | | |
|  |  | | | | | | | | | | | | | | | | | | | | | | |
| **Stage** |  | | | | | | | | | | | | | | | | | | | | | | |
| - LN mets | Yes |  | | |  | | | No | | |  | | | |  | | | | | | | | |
| - Distant mets | Yes |  | | |  | | | NO | | |  | | | Sites(if yes) | | | | | | 1.  2. | | | |
|  |  | | | | | | | | | | | | | | | | | | | | | | |
| **Treatment** |  | | | | | | | | | | | | | | | | | | | | | | |
| - Surgery | Yes | | |  | | |  | | | | | | No | | | | | |  | | | | |
| - Type of surgery | If Yes: | | | | | | | | | | | | | | | | | | | | | | |
|  |  | | | | | | | | | | | | | | | | | | | | | | |
| Radiotherapy | Yes | | |  | | |  | | | | | | No | | | | | |  | | |  | |
| - Intent | Radical | | |  | | |  | | | | | | Palliative | | | | | |  | | |  | |
| - Site | If Yes: | | | | | | | | | | | | | | | | | | | | | | |
|  |  | | | | | | | | | | | | | | | | | | | | | | |
| Systemic therpay | Yes | |  | | |  | | | | | | No | | | | |  | | | | |  | |
| Types | If Yes: | | | | | | | | | | | | | | | | | | | | | | |
|  |  | | | | | | | | | | | | | | | | | | | | | | |

| RAI | Yes |  |  | NO |  |
| --- | --- | --- | --- | --- | --- |
